# Genome-wide identification and expression pattern analysis of the ribonuclease T2 family in *Eucommia ulmoides*

**DOI:** 10.1038/s41598-021-86337-5

**Published:** 2021-03-25

**Authors:** Jun Qing, Qingxin Du, Yide Meng, Panfeng Liu, Hongyan Du, Lu Wang

**Affiliations:** 1 Non-Timber Forest R&D Center, Chinese Academy of Forestry, Zhengzhou, 450003 China; 2 Eucommia Engineering Research Center of State Forestry and Grassland, Zhengzhou, 450003 China; 3 Key Laboratory of Non-Timber Forest Germplasm Enhancement & Utilization of State Forestry and Grassland Administration, Zhengzhou, 450003 China

**Keywords:** Computational biology and bioinformatics, Developmental biology, Genetics, Molecular biology, Plant sciences, Structural biology

## Abstract

The 2′,3′-cycling ribonuclease (RNase) genes are catalysts of RNA cleavage and include the RNase T2 gene family. RNase T2 genes perform important roles in plants and have been conserved in the genome of eukaryotic organisms. In this study we identified 21 *EURNS* genes in *Eucommia ulmoides Oliver (E. ulmoides)* and analyzed their structure, chromosomal location, phylogenetic tree, gene duplication, stress-related cis-elements, and expression patterns in different tissues. The length of 21 predicted EURNS proteins ranged from 143 to 374 amino acids (aa), their molecular weight (MW) ranged from 16.21 to 42.38 kDa, and their isoelectric point (PI) value ranged from 5.08 to 9.09. Two classifications (class I and class III) were obtained from the conserved domains analysis and phylogenetic tree. EURNS proteins contained a total of 15 motifs. Motif 1, motif 2, motif 3, and motif 7 were distributed in multiple sequences and were similar to the conserved domain of RNase T2. *EURNS* genes with similar structure and the predicted EURNS proteins with conserved motif compositions are in the same group in the phylogenetic tree. The results of RT-PCR and transcription data showed that EURNS genes have tissue-specific expression and exhibited obvious trends in different developmental stages. Gene duplication analysis results indicated that segment duplication may be the dominant duplication mode in this gene family. This study provides a theoretical basis for research on the RNase T2 gene family and lays a foundation for the further study of *EURNS* genes.

## Introduction

The 2′,3′-cyclizing ribonucleases (RNases) genes are catalyst of RNA cleavage. They are ubiquitous components of cells and play crucial roles in the regulation of vital processes in cellular RNA metabolism^1^. Ribonucleases are usually secreted or targeted at membrane-binding regions such as lysosomes or vacuoles^2^. The 2′3′ cycling RNases proteins play a crucial rule in all organisms and are mainly divided into RNaseT1, RNase T2 and RNase A^3^. The RNase T1 genes are widespread in fungi and bacteria whereas RNase A genes are always found in animals. RNase T2 are distributed in all livings organisms, including humans, animals, plants, bacteria, fungi and viruses^1^. By comparing the primary sequences of RNase T2, two common peptides (CAS I and CAS II) have been nominated as conserved active site fragments in all enzymes.

RNase T2 were conserved in the genome of all plants and perform an important role with functions that have been maintained during evolution^1, 4^. Plant RNase T2 genes are divided into class I, class II, and class III based on sequence similarities, and gene structure such as gene intron number and position^5, 6^. The class I proteins are related to a variety of stress responses and there are obvious diversifications in gene classification and evolution that cause their numbers to be unstable in different species^7^. Class I proteins are highly expressed in some physiological processes, such as the development and senescence of xylem cells. RNase LX (S-like RNase) is contained in Class I in tomato and is specifically expressed during endosperm mobilization and leaf and flower senescence^8^. Class II proteins are relatively conservative and are a single or a few genes are present in each plant genome^7^. According to their conservation and gene expression, class II RNases have a housekeeping role in plants. This role is thought to be the ancestral function of RNase T2 enzymes^4, 9^. RNaseLER enzyme, one of class II member of tomato, shows preferential expression in guard cells^10^. Plants that lack RNS2 activity exhibit a constitutive autophagy phenotype, indicating that RNS2 belongs to class II and is essential for maintaining cellular homeostasis^11, 12^. Class III proteins are mainly distributed in core eudicots, unlike class I and II proteins, which are distributed in all terrestrial plants^13^. Class III RNases are believed to be S-RNase, which plays a key role in the recognition and rejection of self-pollination in the self-incompatibility system in Solanaceae, Rosacea, and Plantaginaceae^14–16^. For example, in apples and *Citrus grandis* var*. Shatianyu* Hort, S-RNase plays a central role in rejecting self-pollination^17, 18^. The class III RNase group also includes genes that are structurally similar to S-RNases and non S-RNases, but without participation in the self-incompatibility system^5, 6^. Therefore, according to the function, the plant RNase T2 family was also classified as belonging to S-RNase and S-like RNase subfamilies^19^. The only clearly established, specialized function of S-RNase is associated with gametophytic self-incompatibility^18, 20–22^. The S-like RNases participate in defense responses and metabolism. They are known to be associated with phosphate starvation^23–26^, inhibits hyphal growth^27^, senescence^8, 28^, programmed cell death^29^ and reponse to pathoges^30^. In addition to playing a role in self-incompatibility, S-RNases may also play a defensive role. Petunia S-RNases which have characteristics between S- and S-like RNase, were expressed in nectar^31, 32^.

*Eucommia ulmoides* Oliver (*E. ulmoides*) has high industrial and medicinal value. It is a Tertiary relict plant and endemic to China. *E. ulmoides* is the sole living species of the *Eucommia* genus and belongs to the Eucommiaceae family^33^. The increasing numbers of sequenced genomes has facilitated the evolutionary studies of the gene family, and genome-wide analysis of RNase T2 genes have been described in many different species. This study would be helpful to understanding their evolutionary origin and biological functions and provide a basic for the classification and functional identification of RNase T2 gene of* E*. *ulmoides*, a dioecious plant.

## Result

### Identification of the RNase T2 proteins

We identified 21 genes corresponding to the RNase T2 family (*EURNS*) in the *E. ulmoides* genome annotation and comfirmed the sequences by cloning experiment. Detailed information is shown in Table 1 (sequences showed in Table S2). The *EURNS* gene lengths ranged from 429 to 1125 bp and cellular localization predictions suggest that they are distributed among the nuclear, cytoplasmic, mitochondrial, plasma membrane, and extracellular domains. *EURNS3* was in the mitochondrial domain, *EURNS18* and *EURNS23* were in the plasma membrane, *EURNS1* and *EURNS2* were in the cytoplasmic, six genes were in the nuclear domain and ten genes were in the extracellular domain. The prediction of subcellular localization indicated the diversity of RNASET2 gene distribution. The results showed that the length of any particular gene was not related to its location.

**Table 1:** Detailed information on *EURNS* genes.

Gene name	Gene ID	Coding sequence length (bp)	Exons number	Introns number	Location			
					Subcellular location	Sacffold name	Start position	End position
EURNS1	EUC00095-RA	594	2	1	Cytoplasmic	scaffold888_obj (−)	62447	65335
EURNS2	EUC00098-RA	814	3	2	Cytoplasmic	scaffold888_obj (+)	84553	86293
EURNS3	EUC00103-RA	1026	3	2	Mitochondrial	scaffold888_obj (−)	107725	109081
EURNS4	EUC00105-RA	729	2	1	Nuclear	scaffold888_obj (+)	122317	123196
EURNS5	EUC00106-RA	429	2	1	Extracellular	scaffold888_obj (+)	160118	160573
EURNS6	EUC01560-RA	798	2	1	Extracellular	scaffold2044_obj (−)	11489	12502
EURNS7	EUC02920-RA	1083	3	2	Extracellular	scaffold956_obj (−)	18187	19312
EURNS8	EUC02958-RA	702	2	1	Nuclear	scaffold180_obj (+)	11635	18494
EURNS9	EUC06672-RA	660	2	1	Nuclear	scaffold1037_obj (+)	144412	146148
EURNS10	EUC07851-RA	1095	7	6	Extracellular	Super-Scaffold_97 (−)	1174925	1176089
EURNS11	EUC12186-RA	717	2	1	Extracellular	Super-Scaffold_57 (+)	1761911	1762922
EURNS12	EUC12480-RA	1125	3	2	Extracellular	Super-Scaffold_57 (+)	2501510	2503004
EURNS13	EUC15884-RA	723	2	1	Nuclear	scaffold175479_obj (+)	707	1874
EURNS14	EUC18398-RA	684	2	1	Nuclear	scaffold807_obj (−)	60605	62196
EURNS15	EUC18399-RA	714	2	1	Extracellular	scaffold807_obj (−)	100409	101229
EURNS18	EUC19340-RA	672	2	1	PlasmaMembrane	scaffold263_obj (+)	502877	510626
EURNS19	EUC22472-RA	681	4	3	Extracellular	scaffold207_obj (+)	265387	269823
EURNS20	EUC23994-RA	732	2	1	Extracellular	scaffold1086_obj (+)	19370	20190
EURNS21	EUC23999-RA	681	4	3	Extracellular	scaffold1086_obj (+)	69616	71086
EURNS22	EUC24001-RA	744	2	1	Nuclear	scaffold1086_obj (+)	158279	159198
EURNS23	EUC25858-RA	1119	10	9	PlasmaMembrane	scaffold1187_obj (−)	27342	28503

### Sequences structure and motif composition of *EURNS*

Sequences structure analysis included analysis of exon–intron organization, length of CDS (coding sequences) and predicted proteins, protein molecular weight (MW), isoelectric points (PI) and the secondary structure of predicted protein (Table 1 and Table S1). The exon–intron organization is shown in Table 1. Thirteen genes with two exons, four genes with three exons, two genes with four exon and only one gene with ten exons were identified. Among 21 predicted EURNS proteins (Table S1), EURNS12 was determined to be the longest protein with 374 amino acids (aa) while the shortest one was *EURNS*4 with 143 aa. The MW of the proteins ranged from 16.21 to 42.38 kDa, whereas the pI ranged from 5.08 (*EURNS*21) to 9.09 (*EURNS*12). The secondary structure of the protein sequence was analyzed to predict the alpha helix, beta turn, and grand average of hydropathicity (GRAVY). The result are shown in Table S1. The value of the alpha helix, beta turn, and GRAVY ranged from 35 to 145, 8 to 45, and − 0.681 to 0.04, respectively. These values varied greatly in predicted protein sequences and secondary structures, which may be related to the function of the proteins.

**Figure 1:**
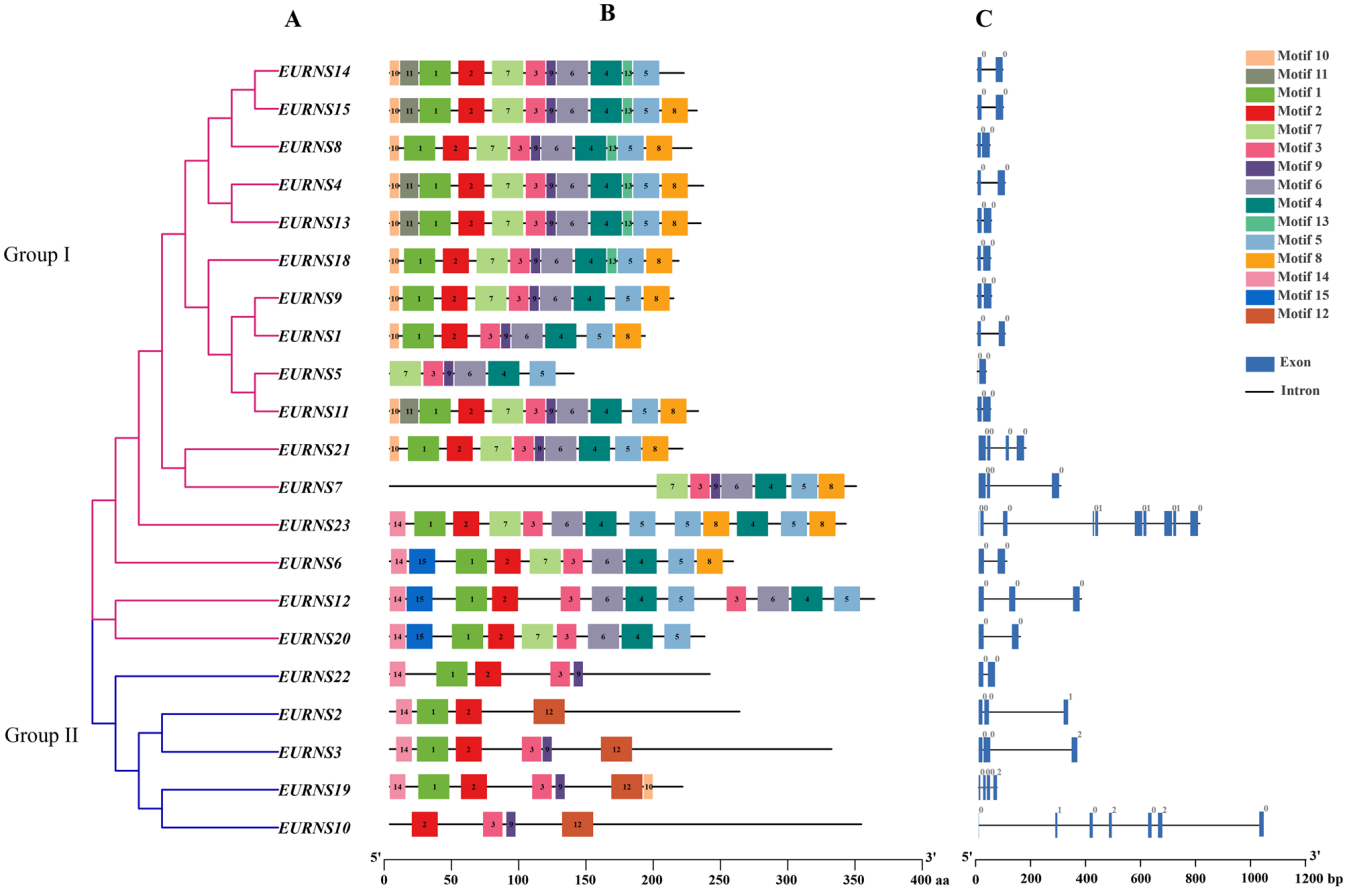
Phylogenetic relationships, architecture of conserved protein motif and gene structure of *EURNS* genes. (**A**) The phylogenetic tee was constructed based on the full-length of predicted proteins using MAGE X software. (**B**) The motif composition of EURNS proteins. Fifty motifs are displaced in different colored boxes, detail information showed in Figure S1. (**C**) Exon and intron structure of *EURNS* genes. Blue boxes indicates the exon; black lines indicate introns. The number indicates the phases of corresponding introns.

We identified the conserved motifs in predicted EURNS amino acid sequences using the MEME program with default parameters. Fifty conserved motifs were found in 21 EURNS members. The results are shown in Fig. 1 and motif information is shown in Figure S1. The motif length ranged from 8 amino acids to 25 amino acids, and the number of motifs varied in EURNS sequences.

The motif 1, motif 2, motif 3 and motif 7 were distributed in multiple sequences and were similar to the conserved domain of RNase T2. In particular, motif 2 and motif 3 corresponded to CAS I and CAS II and they were considered to be a conservative motif in predicted EURNS proteins. MUSCLE multisequence alignment of predicted EURNS protein sequences was used to construct a phylogenetic tree with ML (maximum likelihood method). The EURNS proteins can be divided in two group in by combining motif distribution and phylogenetic tree (see below). group I was not only dominated by four conserved motifs (motif 1, motif 2, motif 3, and motif 7) but also by motif 4, motif 5, motif 6, motif 7, and motif 8, and some sequences contained motif 9 and motif 10. In addition to two conserved motifs (motif 2 and motif 3), most group II proteins included at least two of the following four motifs: motif 1,motif 9, motif 12, and motif 14. Some sequences in group I and group II contained at least two of four conserved motifs. In Fig. 1, it is evident that both *EURNS5* and *EURNS7* lack motif 2, and *EURNS2* lacks motif 3. Interestingly, these genes are more readily available in cloning experiments than others. In conclusion, EURNS genes with similar structures and the predicted EURNS proteins with conserved motif compositions are in the same group of the phylogenetic tree. These findings strongly supported the credibility of the group classification.

### Genome distribution and gene synteny analysis of *EURNS* genes

**Figure 2:**
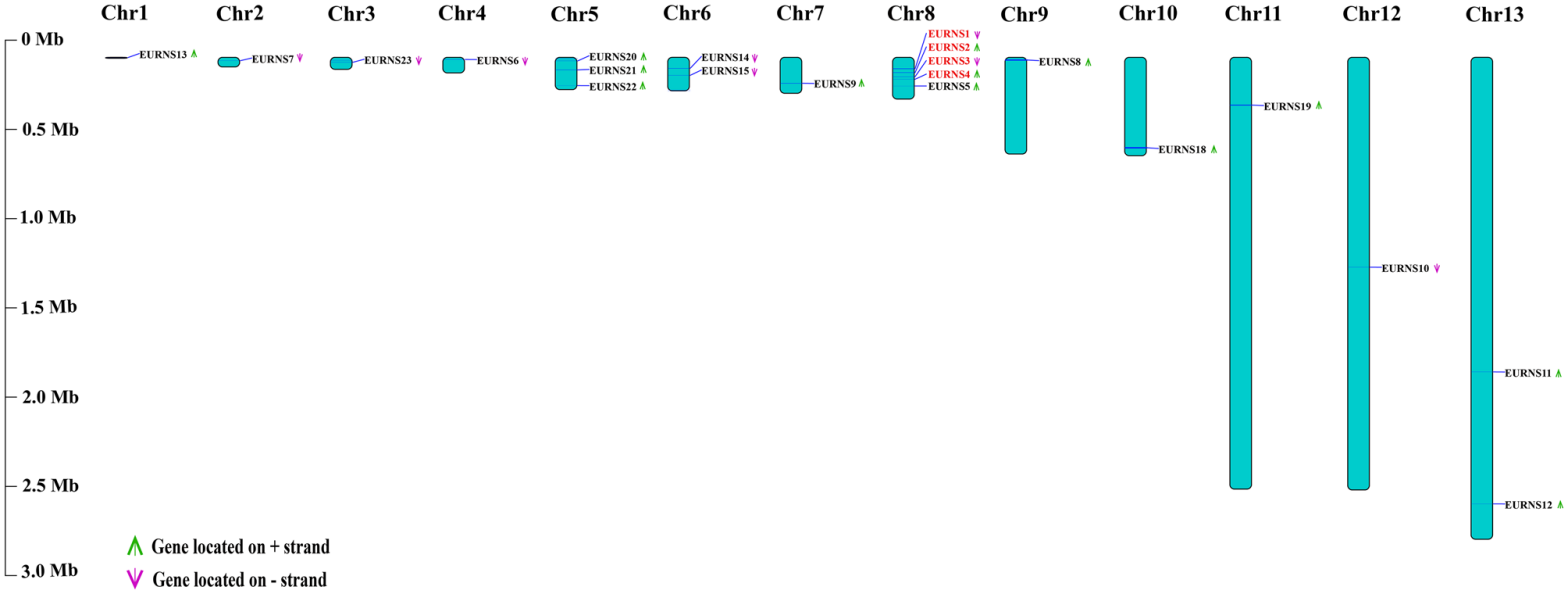
The chromosome distribution of *EURNS* genes. Only chromosomes contained *EURNS* genes are represented in figure. The chromosome number is indicated at the top and the length label in the left. The tandem duplication genes are in red color. The arrows next to genes shows the direction of the transcription.

The genomic location of *EURNS* genes were obtained from genomic data listed in Table S3. All *EURNS* genes were scattered on thirteen chromosomes (Chr: the chromosome is indicating scaffold in *E. ulmoides*) as shown in Fig. 2. *EURNS1*–*EURNS5* members were mapped on chr8, *EUENS20–EUENS22* on chr5. *EUENS14–EUENS15* were located on chr6 and *EURNS11-EURNS12* on Chr13, other genes were corresponded to one chromosome. As seen in previous studies, there was no positive correlation between the scaffold length and the number of genes. A chromosomal region within 200 kb containing two or more genes was defined as a tandem duplication event^34^. Genome duplication events are mainly based on tandem and segmental assignments and occur during plant evolution^35, 36^. In our study, four *EURNS* genes (*EURNS1 and 2, EURNS3 and 4*) were found with tandem duplication events on chr8 (Fig. 2 and Table S4). In addition to the tandem duplication events, segmental duplication with seven *EURNS* genes were identified with BLASTP and MCScanX methods (Figure S2 and Table S4). These results indicated that some *EURNS* genes may have been generated by gene duplication and that duplication events were a crucial driving force in *EURNS* evolution.

To further infer the phylogenetic mechanisms of the RNase T2 family, we constructed four comparative syntenic maps of *E. ulmoides* with three dicots (Arabidopsis, grape, and tomato) and one monocot (rice) that are shown in Figure S3. All syntenic gene pairs contained *EURNS10*. The comparative map between *E. ulmoides* and grape not only contained *EURNS10* but also contained *EURNS19* (Figure S3 and Table S5 (1)), which may indicate that *EURNS10* existed before the ancestral divergence whereas *EURNS19* may have formed after the divergence of dicot-monocot plants.

### Phylogenetic analysis and classification of predicted EURNS proteins

**Figure 3:**
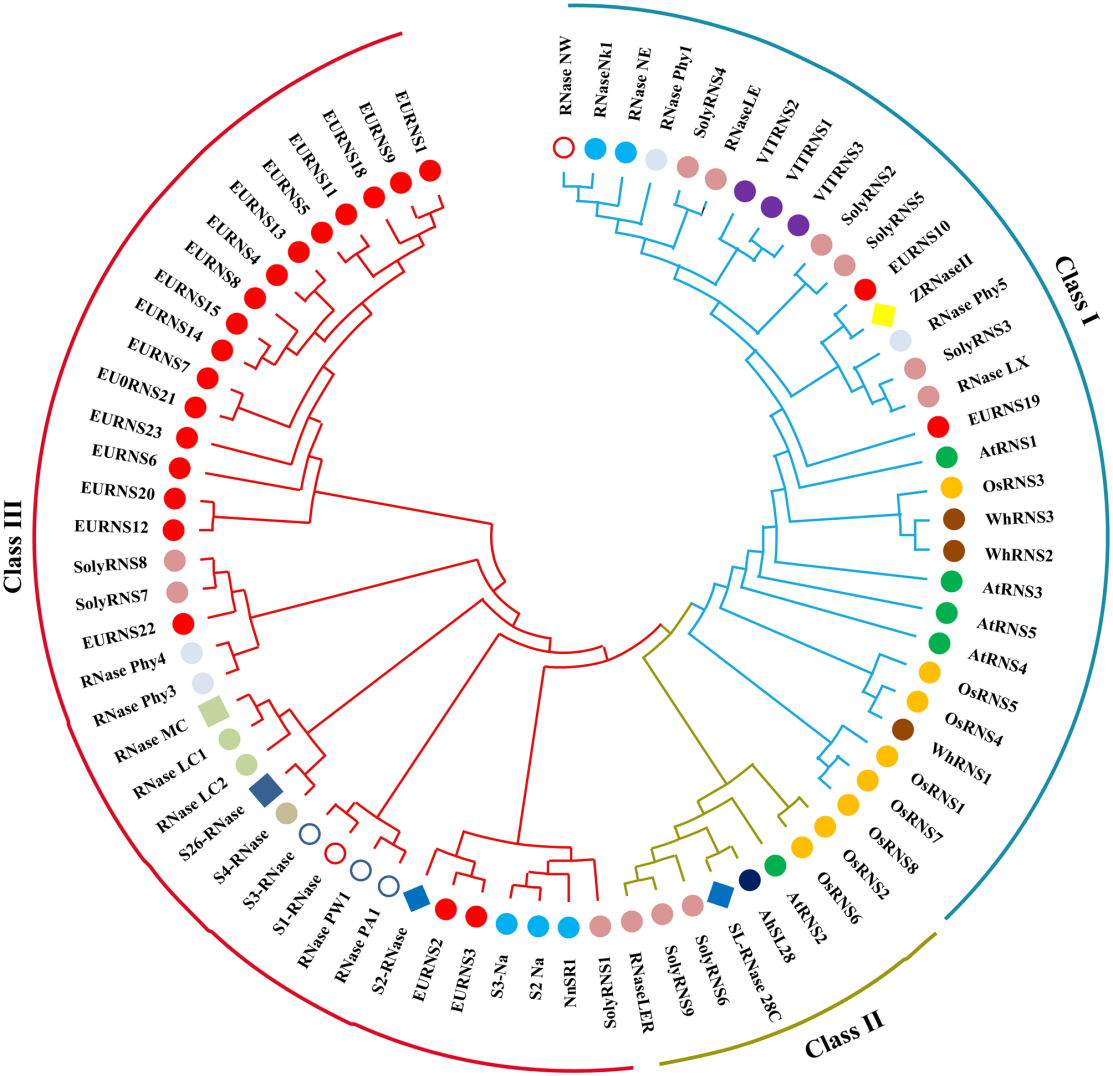
Phylogenetic tree and classification of predcted EURNS proteins. Please refer to Table S5 (2) for the source and accession number of the protein sequence. Maximum Likelihood was created by MEGA X with Muscle multiplied alignment with default parameters.

The function of S-RNase, which plays a central role in self-incompatibility, has been studied in detail in many flowering plants^1, 13, 18^. S-like RNase is not active in self-pollen recognition but is widespread in responses to abiotic and biotic stress . There are three groups of plant T2 RNases: class I, class II, and class III^10^, with class I and II corresponding to S-like RNases, while most of S-RNases are included in class III. The study of some S-like RNase genes has not yielded clear results on their classification. We used the annotated RNase T2 proteins from two monocots (rice and wheat) and three dicots (Arabidopsis, tomato, and grape), and some typical S-like and S-RNase proteins using the multiple sequence comparison by log expectation (MUSCLE) multiple alignment method to construct a phylogenetic tree (Fig. 3 and Table S5 (2)). The classification of the EURNS members was based on previous studies on RNase T2 proteins^9, 10, 13, 31^. The EURNS members were distributed in two groups: class I and class III. EURNS10 and EURNS19 were clustered with class I members and other members, the largest proportion, were clustered alone separately closer with a hybrid between S-like and S-RNases. These results also corroborated the previously proposed classification of the RNase T2 proteins^9^. In phylogenetic tree, except EURNS2/3, the other EURNS are mainly clustered with Phy3 and Phy4 (Fig. 3) which have characterisics between S-RNase and S-like and are expressed in nectar^32^. The class III proteins in *E ulmoides* are likely not involved in self incompatibility and may have some other functions that need further study.

### Transcriptional profile and RT-PCR analysis of *EURNS*

To investigate the expression patterns of *EURNS* genes in various tissues, RNA-seq data were downloaded from NCBI. Six different tissues and one salt treatment were included in the analysis. The fragments per kilobase of transcript per million mapped reads (FPKM) data were used to analyze the spatiotemporal *EURNS* genes expression in *E. ulmoides* (Figures S4–5). Five genes (*EURNS2, 3, 5, 7* and *12*) were not expressed in any sample. These may be pseudogenes or have special temporal and spatial expression patterns that were not captured in these datasets. Two *EURNS (EURN9/18)* genes were expressed in all detected samples and may have a housekeeping role in the development of *E. ulmoides*. In particular, the expression level of *EURN18* was relatively stable throughout the growth and development process, and was highly expressed compared with other genes, further supporting its potential role as a housekeeping gene. Some genes exhibited obvious trends in different development stages.

Although, *EURNS8, EURNS10* and *EURNS19* were expressed during fruit and leaf developmental stages, *EURNS8* and *EURNS19* almost no expression in bark. The irregular expression of *EURNS10* in leaves and flower buds also suggests that some genes are tissue-specific. In addition, the expression of genes under salt treatment was investigated in roots and the results showed that the expression of some genes is associated with salt treatments. Under salt treatments, the expression of *EURNS4, EURNS8* and *EURNS10* increased, while the expression of *EURNS*6 and *EURNS19* decreased. In the phylogenetic tree analysis, *EURNS10* and *EURNS19*, which are related to a variety of stress responses, were classified into class I. This is further validated our phylogenetic tree results.

To validate the expression pattern of the *EURNS* genes in different tissues, the expression levels of 10 randomly selected genes from RNase T2 gene family from different phylogenetic classes were analyzed. Expression levels were detected by using quantative Reverse Transcription-Polymerase Chain Reaction (RT-PCR) in five tissues. The primer of the 10 selected *EURNS* genes are listed in Table S6. The result showed that *EURNS* genes have tissue-specific expression and all ten genes were detected in the tissues analyzed (Figure S6 and Table S8). Although *EURNS1*0 and *EURNS18* genes were not in same classification group, they were expressed significantly higher in fruit and they may be associated with fruit development. In transcriptome analysis, expression of *EURNS10* was changed during the fruit development stage that also confirms the RT-PCR data reliability.

### The *cis-regulatory* elements analysis of *EURNS*

Species specificity is generally considered to be the production of species-specific proteins, but many homologous proteins display difference in their expression that contribute to phenotypic divergence^37^. Regulatory evolution contains pervasive *cis-*regulatory elements (for example, enhancers)^38^. Transcription factors (TF) regulate plant development and physiology by responding to hormones and regulating gene expression^38^. TFs can control expression of target genes by binding to response elements (REs/cis-regulatory elements) in regulatory promoter regions^39^. Gene expression is controlled by both cis- and trans-regulatory factors, and mutations in either type can alter expression. *Cis*-regulatory differences account for a greater proportion of expression differences among species than within species. *Cis*-regulatory DNA sequences contain binding sites that interact with diffusible trans-regulatory proteins and RNA^37, 38^.

The PlantCare software was used to analyse the *cis-regulatory* elements in *EURNS* promoters. In *E. ulmoides*, a great diversity of regulatory elements were predicted in promoter regions of *EURNS* genes. We summarized the most common elements related to light response (ACE, LAMP-element, AE-box, I-box, ATCT/GT1/ TCCC/TCT-motif, Box 4, G-Box, sp1 and MRE) , hormone response (TGA-element, P-box, TATC-box, CGTCA/ TGACG-motif, TGA-element, ABRE), stress response (ARE, GC-motif, LTR, MBS, TC-rich repeats and WUN-motif), development regulation (RY-element, HD-Zip 1, GCN4_motif and CAT-box) and biosynthesis regulation (MBSI, O2-site), showed in Table S7 and Figure S7. The discovery of various *cis-regulatory* elements in these promoters illustrated that the *EURNS* genes might be involved in multiple developmental and stress response pathways.

## Discussion

RNase T2 proteins are conserved in plant genomes and play an important role in these organisms^1, 4^. The RNase T2 family has been studied in many plants, but not in *E. ulmoides*, a dioecious plant. In our study, we identified 21 genes belonging to RNase T2 family in this species. Comparison the number of RNase T2 genes of *E. ulmoides* with other sequenced plant genomes showed *E. ulmoides* has a greater number of genes suggesting a great degree of diversification for the RNase T2 family in plants^7, 9, 10, 12^. Although *EURNS* members are only distributed in two class, our results are also consistent with previous phylogenetic studies^1, 13, 40^. Class I and class II correspond to S-like RNases, while Class III may correspond to S-RNases and non S-RNases. Generally, class III proteins are considered S-RNase and have been shown to be related to self-incompatibility in monoecious species^22, 40, 41^. Class III proteins in *E. ulmoides* are not likely to be S-RNase because this species is dioecious and has no self-incompatibility system. As class III members account for 90% of the RNase T2 genes in *E. ulmoides*, the specific functions of class III members that do not participate in self recognition need to be further studied. Some studies have shown that class III genes may have evolved from S-RNases but aquired new functions^5, 6^, and some plant class III enzymes were assumed to be related to stress reponses^7, 24^.

Two *EURNS* genes are similar to S-like RNase (class I), although the conservation of amino acid residues that are important for ribonuclease activity and structure varies among them^1, 16^ (Fig. 1). *E. ulmoides* only has two members in class I. Interestingly, all syntenic gene pairs contains *EURNS10* (members of class I). The gene pairs between *E. ulmoides* and grape contained not only *EURNS*10 but also *EURNS19* (both members of class I), which may indicate the *EURNS10* and *EURNS19* might already exist and have some specific function in *E. ulmiodes.* In contrast to class II and class III, the class I proteins had less conserved structure and fewer motifs, which is consistent with the conclusion that class I exhibits diversification in gene classification and evolution^7^. For example, *OsRNS4* seem to have stress response functions, but it has lost its catalytic activity and its specific biological activity is not known^42^. The *EURNS 18* gene was expressed in all samples and may have house-keeping role, which was likely the anscestral function of RNase T2 enzymes^5, 9, 12^. Class I and II proteins are found in all land plants, while Class III proteins are found only in core eudicots^1, 43^. In our study, there were more members of class III found in *E. ulmoides* than in the other species, which may be related to the evolution of *Eucommia*. The specific reasons for this merit further study.

Fifteen different motifs in various arrangements were found among EURNS members. Thus, broad domain shuffling occurred in the protein structures of the EURNS family members. Although,the two genes of class I have less number of motifs, both of them contain motif 2, motif 3, motif 9 and motif 12. Compared with class I, the number of motifs in class III were more diverse. According to the prediction results of predicted EURNS protein, it is found that the isoelectric point (pI) was distributed between 5.08 and 9.09. The isoelectric point distribution of the EURNS protein was also within the scope of previous studies^13^. Expression patterns of all 21 *EURNS* genes in the transcriptome data were diverse. For example, five gene (*EURNS2, 3,5, 7* and *12*) were not expressed in any sample, which may indicate that these are pseudogenes or have special temporal or spatial expression patterns not examined in our study. In contrast, *EURN9* and *18* genes were expressed in all detected samples. The expression of different tissues of selected *EURNS* genes formed three classes in *E. ulmoides*. Their expression in tissue revealed that *EURNS* were expressed in all investigated tissues and organs, and several *EURNS* genes showed tissue-specific expression in different tissues (such as *EURNS10*, *18*, and *23*). Other S-like RNases also show tissue specificity, for example the tissue-specific RNaseLX in the phosphate starvation response was connected with specific RNA turnover processes at the root tip^26^.

TFs may control expression of genes by binding response elements (REs/*cis-regulatory* elements) in promoter regions^39^. Cis-regulated differences account for a greater proportion of expression differences among species than within species. Cis-regulated DNA sequences contain binding sites that interact with diffusible trans-regulatory proteins and RNA. Our predicted results showed that *EURNS* promoters contains a total of 265 cis-regulatory elements. Moreover, we found that genes with high expression levels had a large number of cis-regulatory elements, which suggested that cis-elements play an important role in *EURNS* gene expression. This was consistent with previous research results^38^. Gene duplication events are crucial in genomic rearrangement and often result in the generation of new genes. These duplication events include tandem, segment, and transposition duplication^44^. In *E. ulmoides* RNase T2 family, six genes evolved from tandem duplication and seven genes from segment duplication, indicating that the segment duplication may be the dominant gene duplication on the of this gene family. The remaining genes may have evolved through early divergence or have appeared following gene transposition.

In summary, a genome-wide analysis of the *E. ulmoides* RNase T2 family was performed and 21 *EURNS* genes were identified. Subsequently, analyses of *EURNS* genes on gene structures, phylogeny, chromosomal location, gene duplication, stress-related cis-elements, and expression patterns in different tissues were performed. This research laid the foundation and provided the basis for the study of the RNase T2 gene family in *E. ulmoides*, a dioecious plant.

## Materials and methods

### Identification of *RNase T2 *genes in *E. ulmoides*

The whole genome assembly of the *E. ulmoides* were download from National Center for Biotechnology Information (NCBI, https://www.ncbi.nlm.nih.gov/) with accession number PRJNA357336. Subsequently, the RNase T2 genes of rice (Oryza sativa v7_GIR)^45^, tomato (*Solanum lycopersicum* iTAG2.4)^46^, grape (*Vitis vinifera* Genoscope.12X)^47^ and *Arabidopsis* (*Arabidopsis thaliana* TAIR10)^48^ were all downloaded from Phytozome12 (https://phytozome.jgi.doe.gov/pz/portal.html). Three genes of the RNase T2 gene family in wheat (*Triticum aestivum*) were found in NCBI and downloaded (Table S5: (2)). The Hidden Markov Model (HMM) files corresponding to the Ribonuclease T2 (RNase T2) domain (PF00445) was downloaded from pfam (http://pfam.xfam.org/)^49^ to prepare for identification analysis. HMMER 3.0 was used to scan the RNase T2 genes from the *E. ulmoides* genome database. Default parameters were adopted and the cutoff value was set to 1e-2. The candidate RNase T2 genes that may have contained the RNase T2 domain based on the HMMER 3.0 results were further examined using the NCBI Batch Web CD Search Tool (http://www.ncbi.nlm.nih.gov/Structure/bwrpsb/bwrpsb.cgi) and Simple Modular Architecture Research Tool (http://smart.embl.de/smart/set_mode.cgi). Genes that had no RNase T2 domain and repeated genes were manually deleted. The nomenclature of RNase T2 genes in E. ulmoides were based on the gene ID list in genome data.

Plant materials including leaves (L6), buds (C2), fruit (F6), seeds (S6), and bark (P6) from 6 year-old *E. ulmoides* ‘Huazhong No. 6’ were collected on July 5th 2019 in Yuanyang experimental base of Paulowina Research and Development Center of State Foresty and Grassland Administration and prepare for RNA extraction and qPCR analysis. All tissue samples were immediately frozen in liquid nitrogen and stored at − 80 °C for subsequent analysis. The cloning experiments was used to determine the predicted *EURNS* members in E. ulmoides. Primmers were designed by primmer 5.0 and total RNA was extracted using RNAprep Pure Plant Kit (TIANGEN, Beijing, China) and revesed to cDNA. The PCR cycling conditions were as follows: 94 °C for 3 min, 34 cycles of 94 °C for 10 s, 58 °C for 15 s and 72 °C for 10 s and final extension 72 °C for 5 min.

### Sequences structure and motif composition analysis

To study gene structure, the intron/exon structures for each gene were mapped to their corresponding genes using the online program Gene Structure Display Server (GSDS; http://gsds.cbi.pku.edu.cn)^50^ to compare predicted coding sequences with the corresponding full length sequences. The MEME tool (http://meme.nbcr.net/meme/intro.html)^51^ for protein sequence analysis was used to identify conserved motifs for the candidate *E. ulmoides* RNase T2 proteins. The optimized parameters were employed as follows: the number of repetitions, any; the maximum number of motifs, 10; and the optimum width of each motif, from 6 to 100 residues. The putative isoelectric point (PI) and molecular weight (MW) of the *EURNS* proteins were predicted using Compute PI/Mw (http://web.expasy.org/compute_pi/). The subcellular location of EURAS was predicted using CELLO 2.5 (http://cello.life.nctu.edu.tw/).

### Phylogenetic tree and classification of EURNS proteins

Phylogenetic analysis was based on the whole amino acid sequences. We used species rice, Arabidopsis, tomato, and grape that were explored in the genome study. In addition, three genes of wheat were specially added that were found from NCBI annotation. Proteins from class I–class III, as well as S-RNase and S-Like RNase, have also been added (Table S5:(2)). The OsRNS classification scheme^9^ was used as references to divide the *EURNS* genes into different groups. For the full-length *EURNS* cascade protein sequences, Muscle method was used to conduct multiple alignment for the phylogenetic tree. The freely available software MAGE X^52^ (https://www.megasoftware.net/) using Maximum Likelihood (ML) method with the default parameters was used to construct the phylogenetic tree.

### Analysis of genomic distribution and synteny analysis

All of the identified *EURNS* genes were mapped in their corresponding scaffold on the basis of their physical position. The physical position of all *EURNS* genes and scaffold lengths were obtained from the *E. ulmoides* genome assembly database. The Mapinspect software (http://mapinspect.software.informer.com/) was used to produce the schematic diagrams of the position of *EURNS* genes in scaffolds and manually color the tandem duplication genes. The Multiple Collinearity Scan toolkit (MCScanX) was adopted to analyze the gene duplication events in a LInux environment, with default parameters. To illustrate the syntenic relationships of the orthologous *EURNS* genes obtained from *E. ulmoides* and other selected species, syntenic analysis maps were constructed using the Dual Systeny Plotter software (https://github.com/CJ-Chen/TBtools/releases)^53^. The *E. ulmoides* genome sequences were assembled into scaffolds^33^. In this study we only used eight scaffolds that have orthologous pairs between *E. ulmoides* and other selected species.

### Transcriptional profile analysis and RT-PCR analysis

To analyze the expression of *EURNS* genes, RNA-seq data of leaves, bark, roots, and seeds, and their expression under salt treatment were downloaded from NCBI under the following accession numbers: PRJNA357336 (the developmental stages of fruits, leaves, and bark from April to September), PRJNA321358 (young and mature fruits), PRJNA329457 (salt stress responsiveness), female/male flower buds (SRR2170964 and SRR2170970), seeds (SRR3203241), and the other flower bud expression (unpublished). The transcript abundance of *EURNA* genes was calculated as fragments per kilobase of exon model per million mapped reads (FPKM). The heatmaps were created by HemI1.0 based on the transformed data of log2 (FPKM + 1) values.

The 10 primers were designed by primmer 5.0 and total RNA was extracted using RNAprep Pure Plant Kit (TIANGEN, Beijing, China) following the manufacturer's instructions. A maximum of 1 μg total RNA was used for synthesizing cDNA by HiScript QRT SuperMix (Vazyme, Nanjing, China), and the product was subjected to RT-PCR with an Opticon thermocycler (CFX Connect Real-Time System; Bio-Rad, Hercules, CA, USA) using SYBR Green PCR master mix (Vazyme, Nanjing, China) according to the manufacturer's instructions. The PCR cycling conditions were as follows: 95 °C for 10 min, 37 cycles of 95 °C for 10 s, and 45 °C for 30 s. A 65 °C–95 °C melt curve was analyzed to detect possible primer dimers or nonspecific amplification. Actin and GAPDH were used as the internal controls^54^. RT‐PCR was performed with three replicates per gene. Expression levels were evaluated using the 2∆∆CT method^55^. SPASS 18.0 was used for single factor of variance (OneWay ANOVA) and T test for statistics analysis.

### Promoter *cis-regulatory* elements analysis

The upstream 1500 bp sequence of *EURNS* translation start site was manually cut and submit to the PlantCARE 1.0 ( http://bioinformatics.psb.ugent.be/webtools/plantcare/html) website for prediction. The obtained promoter cis-regulatory elements were manually filtered and plotte using online program Gene Structure Display Server (GSDS; http://gsds.cbi.pku.edu.cn).

## Supplementary Information

Supplementary Information 1.

Supplementary Information 2.

Supplementary Information 3.

## Data Availability

Genome data and transcriptome data of *E. ulmoides* are available at NCBI following accession numbers and UTLs. PRJNA357336: https://www.ncbi.nlm.nih.gov/bioproject/?term=PRJNA357336, PRJNA321358: https://www.ncbi.nlm.nih.gov/bioproject/?term=PRJNA321358, PRJNA329457: https://www.ncbi.nlm.nih.gov/bioproject/?term=PRJNA329457, SRR2170964: https://www.ncbi.nlm.nih.gov/bioproject/?term=SRR2170964 SRR2170970: https://www.ncbi.nlm.nih.gov/bioproject/?term=SRR2170970 SRR3203241: https://www.ncbi.nlm.nih.gov/bioproject/?term=SRR2170970.
