# Supplementary material for: Genome-wide identification and expression pattern analysis of the ribonuclease T2 family in Eucommia ulmoides
Source: Sci Rep. 2021 Mar 25;11:6900. doi: 10.1038/s41598-021-86337-5 (PMC7994793; doi:10.1038/s41598-021-86337-5)
Supplement: Supplementary file 1 — Supplementary Information 1. [file 41598_2021_86337_MOESM1_ESM.docx]

**Genome-wide identification and Expression Pattern Analysis of** **Ribonucleases T2 Family in *Eucommia ulmoides***

**Introduction of Supplementary tables**

Table S1: Detail information of predicted EURNS proteins.

Table S2: List of the 23 identified sequences in this study

Table S3: The location of EURNS genes and Scaffold information

Table S4: Segmental and tandem duplication of *EURNS* genes pairs

Table S5: (1). Syntenic gene pairs between *E.ulmoides* and other four plant species

(2). Corresponding gene ID of species in Phylogenetic tree analysis

Table S6: Primer Information

Table S7: The predicted cis-regulatory elements in the promoter of *EURNS*

Table S8: The raw data of 10 EURNS genes in five tissues corresponding to Figure S6

**Introduction of Supplementary Figures**

Figure S1: Detail information of motif sequences.

The MEME tool ( <http://meme.nbcr.net/meme/intro.html> ) for protein sequence analysis was used to identify conserved motifs for the candidate E. ulmoides RNase T2 proteins.

Figure S2: The synteny analysis of RNase T2 family in *E.ulmoides.*

The word outside of represented the name of Chr and the inner information on the scaffold represented the name of *EURNS* genes. The red line indicates duplication *EURNS* genes pairs. Chr: Chromasome. The Multiple Collinearity Scan toolkit (MCScanX: <http://chibba.pgml.uga.edu/mcscan2/> ) was dopted to analyze the gene duplication events, with default parameters.

Figure S3: Synteny analysis of RNase T2 genes between *E. ulmoides* and four plant species. Gray lines in background indicate the collinear blocks within *E.ulmoide* and other genomes, the red lines highlight the syntenic *RNase T2* gene pairs. The specie name with theprefixs ‘*E.ulmoides’, ‘O.sative’, ‘S.lycopersicum’* ‘*V.vinifera’* and ‘*A.thaliana’* indicate *Eucommia ulmoide*s, *Oryza sativa*, *Solanum lycopersicum*, *Vitis vinifera* and *Arabidopsis thaliana.* The chromosome number indicated at top and bottom of each chromosome with same color. The black font represents name of genes in its places. Syntenic analysis maps were constructed using the Dual Systeny Plotter software ( <https://github.com/CJ-Chen/TBtools> ).

Figure S4: Expression profiles of the *EURNS* genes during development of three tissues.

EuL, EuF and EuB indicate leaf, fruit and bark, respectively. The followed number indicated the date. The clustering tree was constructed by hierarchical clustering using average linkage method.

Figure S5: Expression profiles of *EURNS* genes during development of flower and salt treatment in roots. Eu0h_1 and Eu24h_1 indicated salt treatment CK and 24hours later. BJC and F6 indicate female flower buds, SNJ and M11 indicate male flower buds. Followed number indicated the date. The clustering tree was constructed by hierarchical clustering using average linkage method.

Figure S6: Expression analysis of 10 *EURNS* genes in five tissues. L6, C2, F6, S6 and P6 is represents leaf, flower bud, fruit, seed and sink of fruit, respectively. Note: * indicates significant difference in gene expression in different tissue (P<0.05)；** indicates extremely significant difference in ein gene expression in different tissue (P<0.01).

Figure S7:  Distribution of *cis-regulatory* element in *EURNS* gene family classification of different groups. The upstream 1500bp sequence of *EURNS* translation start site was manually cut and submit to the PlantCARE (<http://bioinformatics.psb.ugent.be/webtools/plantcare/html/>) website for prediction. Finally cis-regulatory elements were manually filtered and plotte using online program Gene Structure Display Server (GSDS; <http://gsds.cbi.pku.edu.cn>).
