# Supplementary material for: Genome-wide identification and expression pattern analysis of the ribonuclease T2 family in Eucommia ulmoides
Source: Sci Rep. 2021 Mar 25;11:6900. doi: 10.1038/s41598-021-86337-5 (PMC7994793; doi:10.1038/s41598-021-86337-5)
Supplement: Supplementary file 2 — Supplementary Information 2. [file 41598_2021_86337_MOESM2_ESM.docx]

Supplementary Figures of Genome-wide Identification and Expression Pattern Analysis of Ribonucleases T2 Family in *Eucommia ulmoides*

Jun Qing, Qingxin Du, Panfeng Liu, Yide Meng, Hongyan Du, Lu Wang*


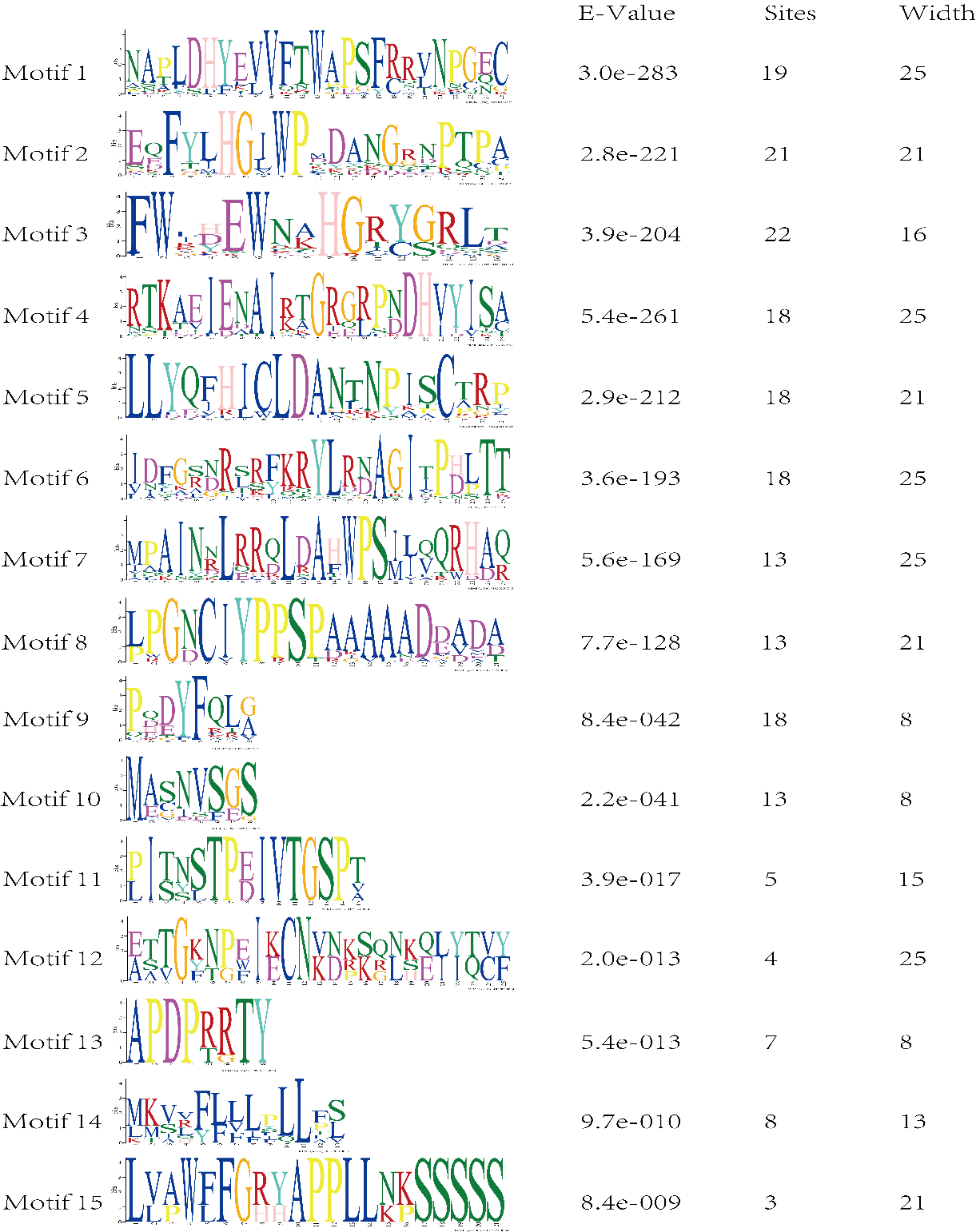


**Figure S1** Detail information of motif sequences.

The MEME tool ( <http://meme.nbcr.net/meme/intro.html> ) for protein sequence analysis was used to identify conserved motifs for the candidate E. ulmoides RNase T2 proteins.


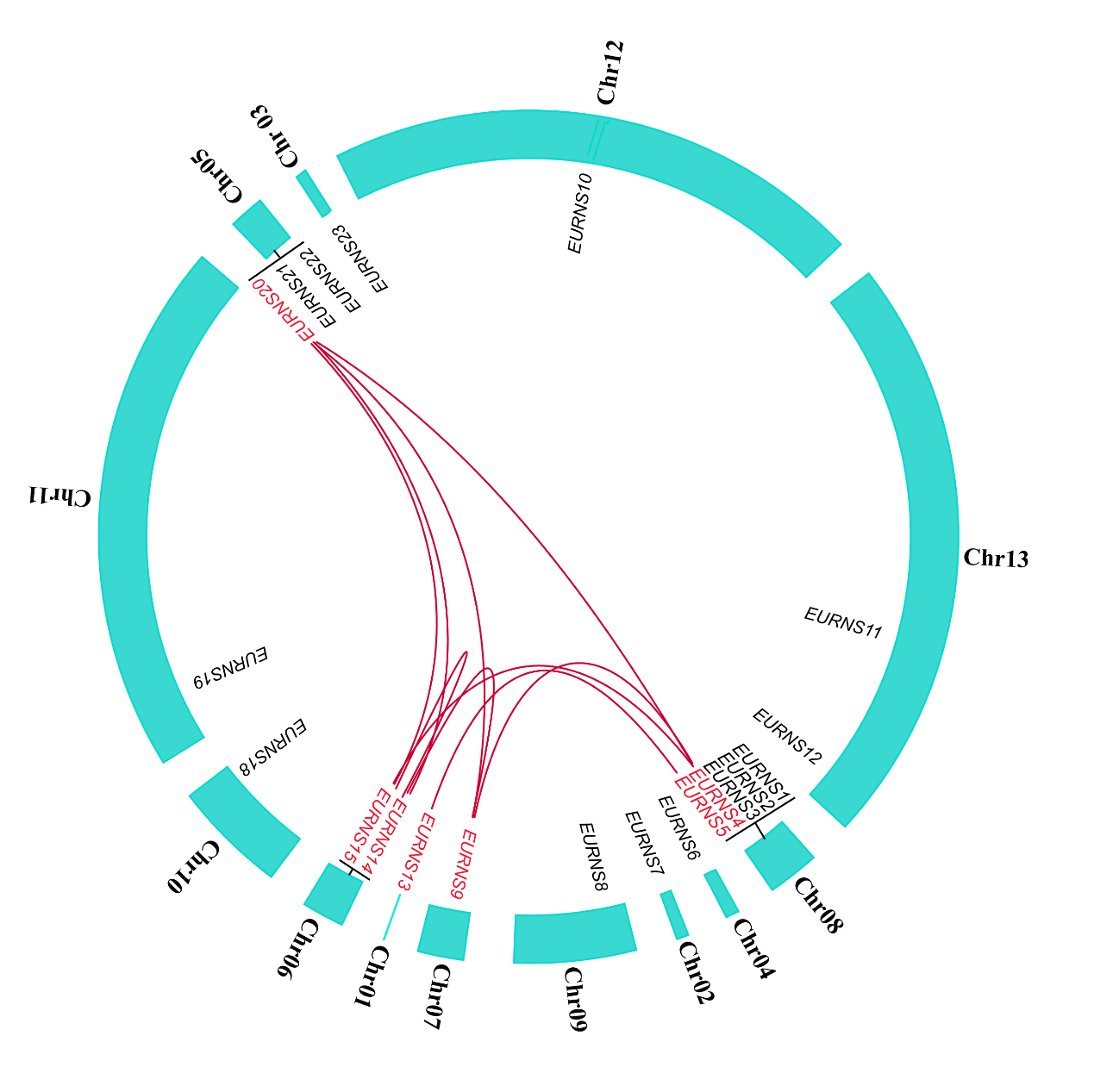


**Figure S2** The synteny analysis of RNase T2 family in *E.ulmoides.*

The word outside of represented the name of Chr and the inner information on the scaffold represented the name of *EURNS* genes. The red line indicates duplication *EURNS* genes pairs. Chr: Chromasome. The Multiple Collinearity Scan toolkit (MCScanX: <http://chibba.pgml.uga.edu/mcscan2/> ) was dopted to analyze the gene duplication events, with default parameters.


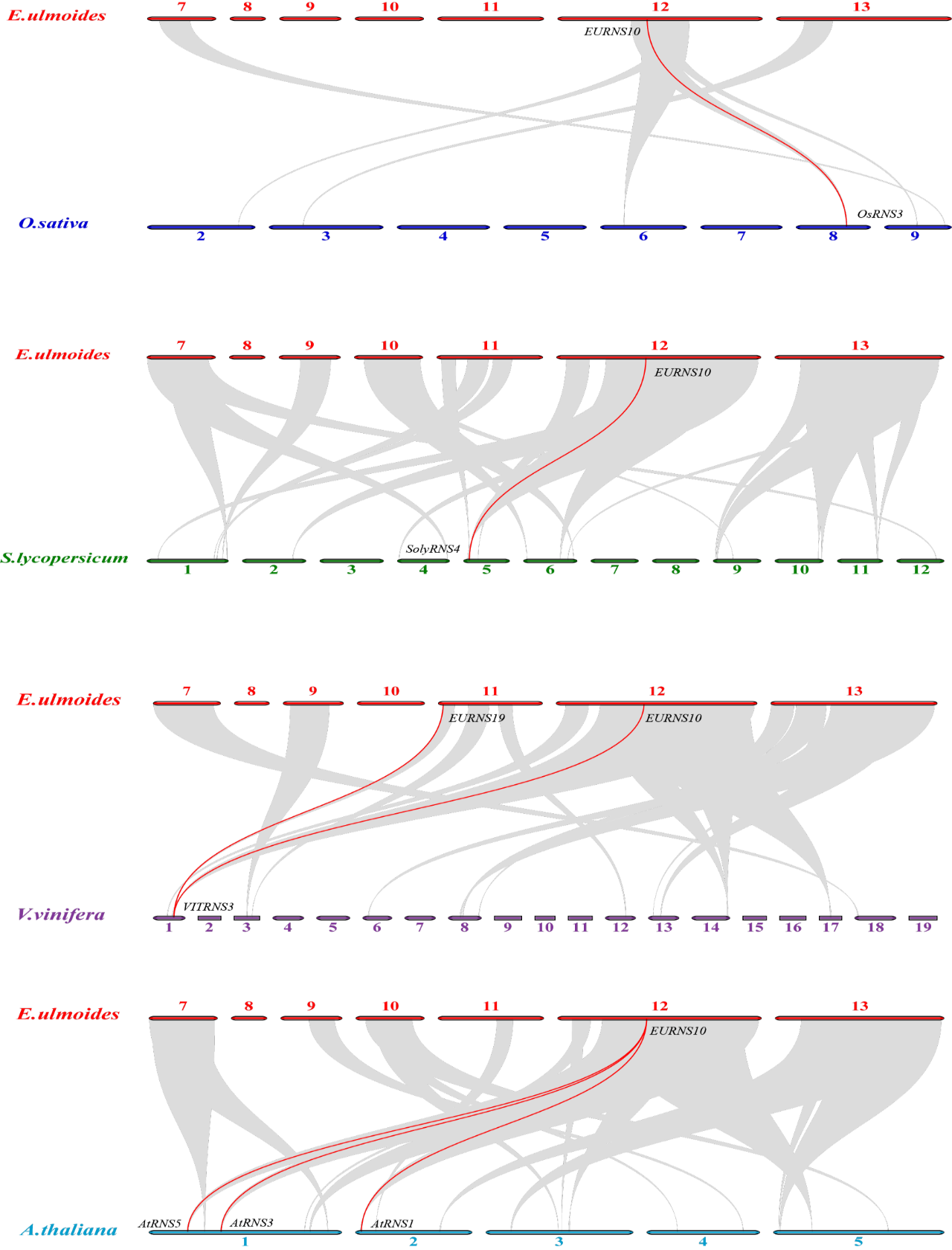


**Figure S3** Synteny analysis of RNase T2 genes between *E. ulmoides* and four plant species. Gray

lines in background indicate the collinear blocks within *E.ulmoide* and other genomes, the red lines

highlight the syntenic *RNase T2* gene pairs. The specie name with theprefixs ‘*E.ulmoides’,*

*‘O.sative’, ‘S.lycopersicum’* ‘*V.vinifera’* and ‘*A.thaliana’* indicate *Eucommia ulmoide*s, *Oryza*

*sativa*, *Solanum lycopersicum*, *Vitis vinifera* and *Arabidopsis thaliana.* The chromosome number

indicated at top and bottom of each chromosome with same color. The black font represents name

of genes in its places. Syntenic analysis maps were constructed using the Dual Systeny Plotter

software ( <https://github.com/CJ-Chen/TBtools> ).


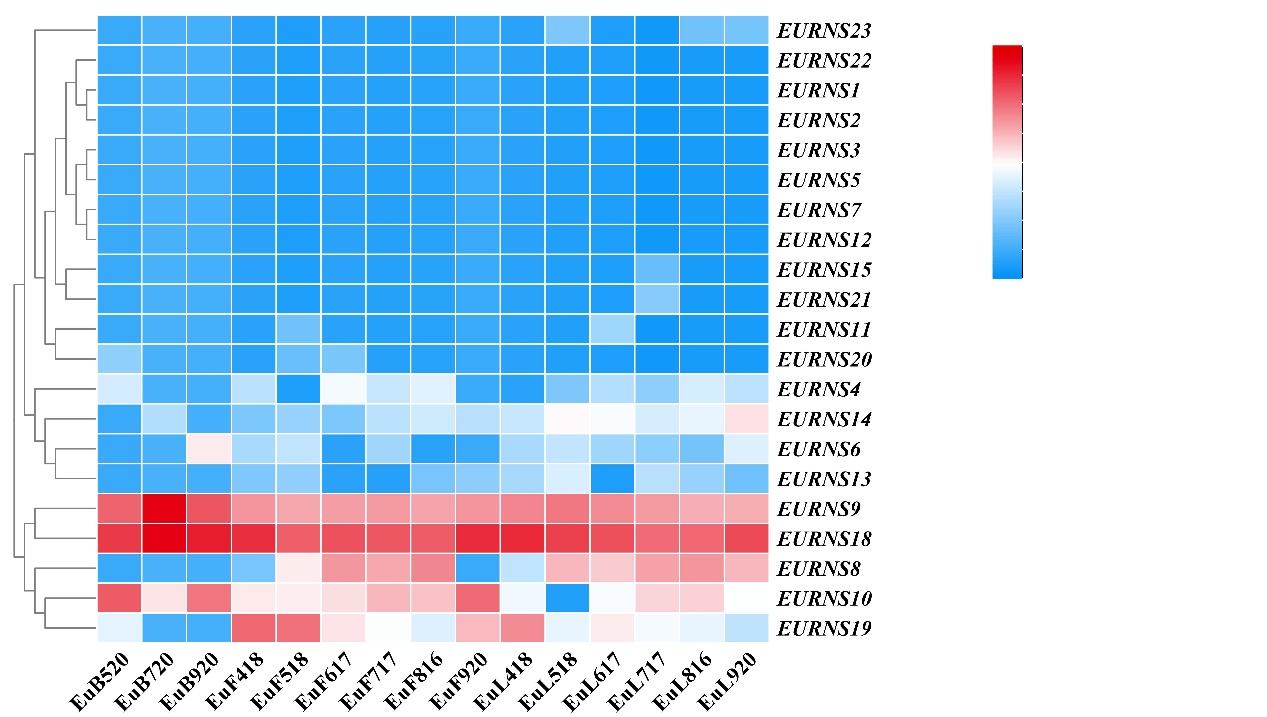


**Figure S4** Expression profiles of the *EURNS* genes during development of three tissues.

EuL, EuF and EuB indicate leaf, fruit and bark, respectively. The followed number indicated the

date. The clustering tree was constructed by hierarchical clustering using average linkage method.


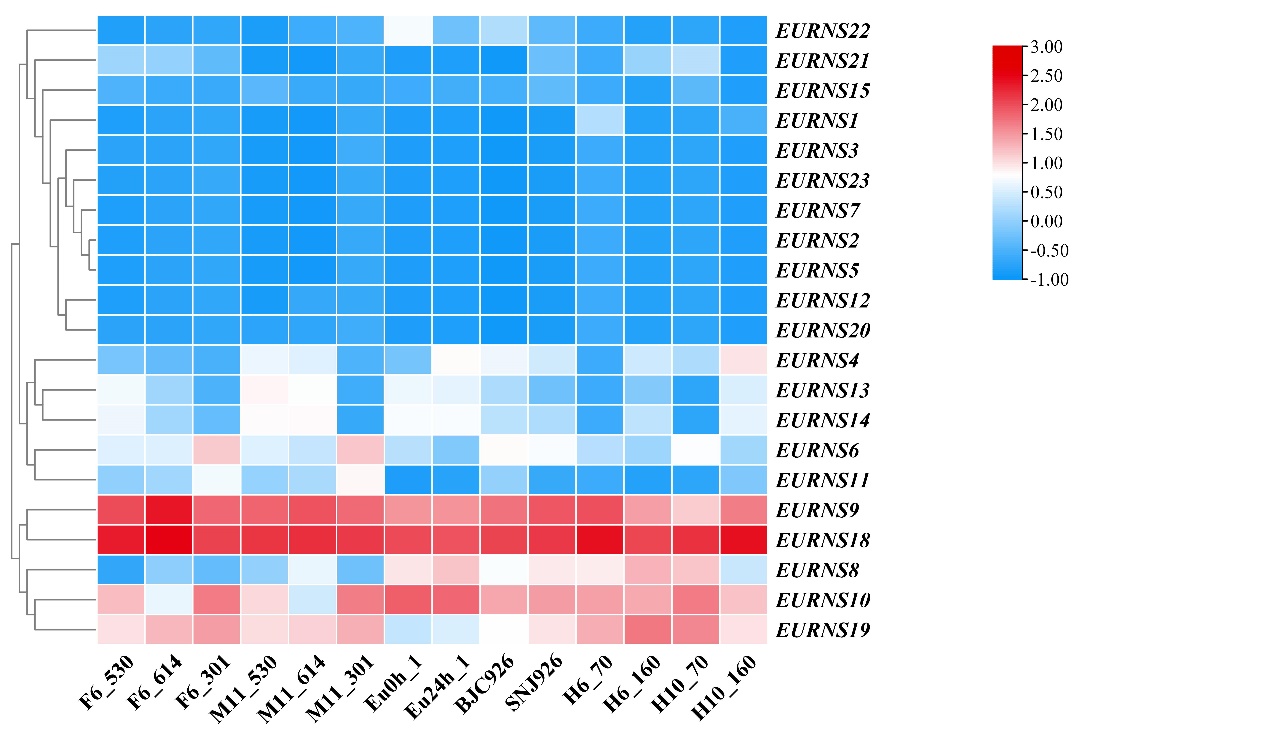


**Figure S5** Expression profiles of *EURNS* genes during development of flower and salt treatment in

roots. Eu0h_1 and Eu24h_1 indicated salt treatment CK and 24hours later. BJC and F6 indicate

female flower buds, SNJ and M11 indicate male flower buds. Followed number indicated the date.

The clustering tree was constructed by hierarchical clustering using average linkage method.


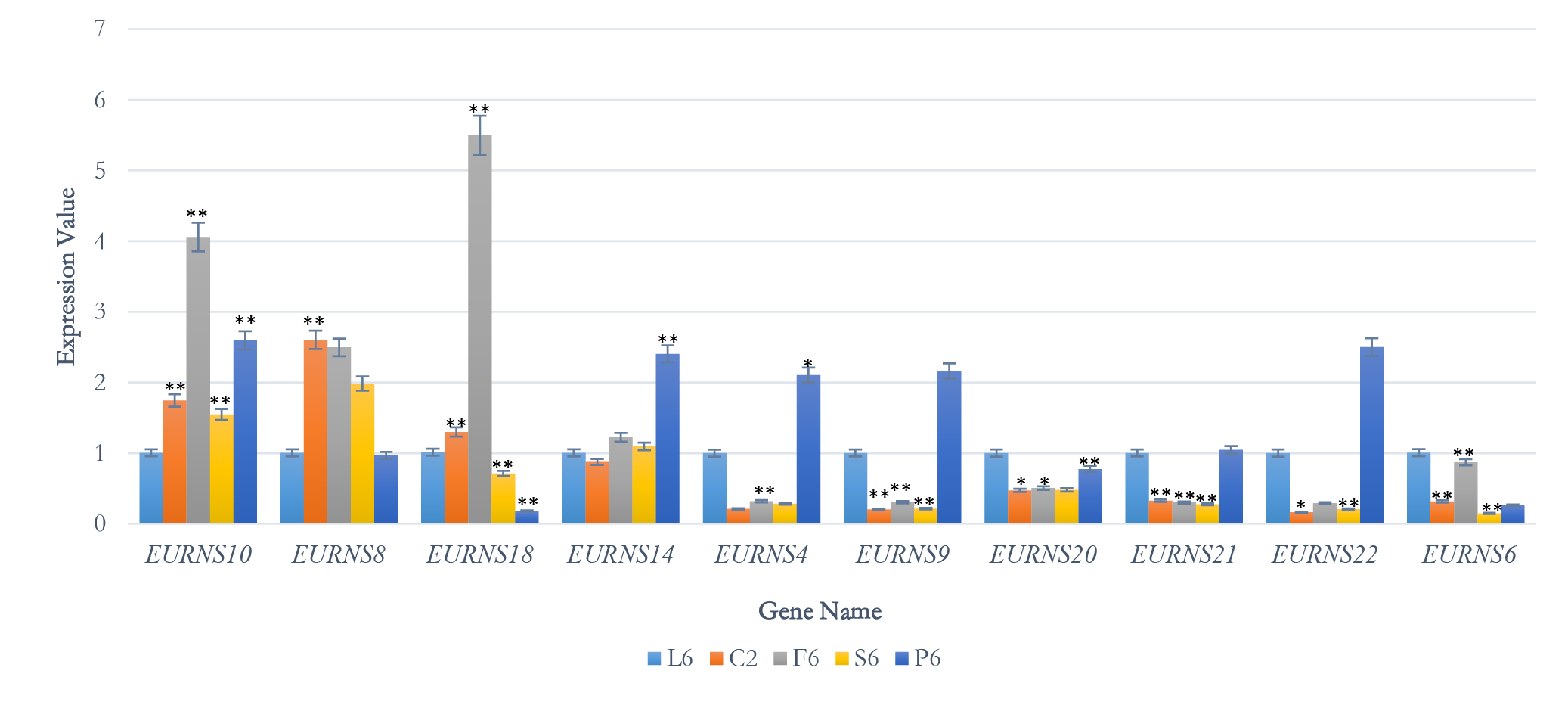


**Figure S6** Expression analysis of 10 *EURNS* genes in five tissues. L6, C2, F6, S6 and P6 is

represents leaf, flower bud, fruit, seed and sink of fruit, respectively.

Note: * indicates significant difference in endogenous hormone content in male and female flower

buds (P<0.05)；** indicates extremely significant difference in endogenous hormone content in

male and female flower buds (P<0.01).


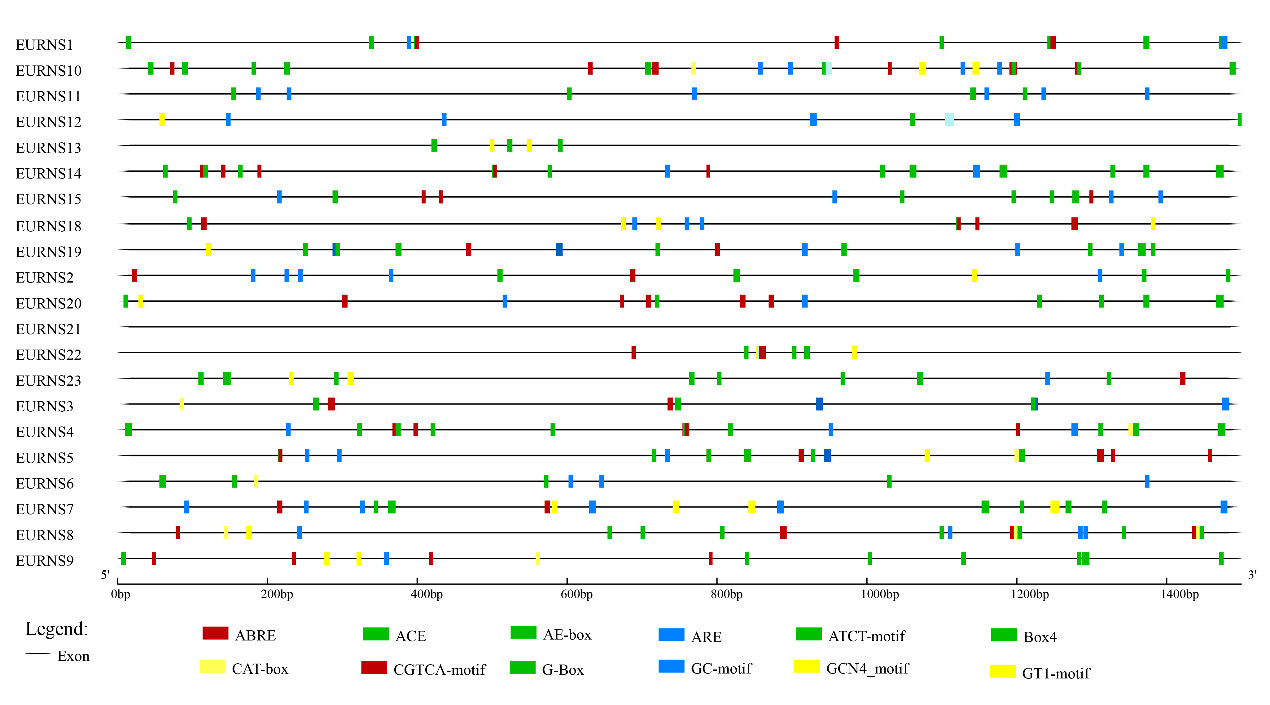


**Figure S7** Distribution of *cis-regulatory* element in *EURNS* gene family classification of different

groups. The upstream 1500bp sequence of *EURNS* translation start site was manually cut and

submit to the PlantCARE (<http://bioinformatics.psb.ugent.be/webtools/plantcare/html/>) website

for prediction. Finally cis-regulatory elements were manually filtered and plotte using online

program Gene Structure Display Server (GSDS; <http://gsds.cbi.pku.edu.cn>).
